# The gut microbiota of insecticide-resistant insects houses insecticide-degrading bacteria: A potential source for biotechnological exploitation

**DOI:** 10.1371/journal.pone.0174754

**Published:** 2017-03-30

**Authors:** Luis Gustavo de Almeida, Luiz Alberto Beraldo de Moraes, José Roberto Trigo, Celso Omoto, Fernando Luis Cônsoli

**Affiliations:** 1 Universidade de São Paulo, Escola Superior de Agricultura “Luiz de Queiroz”, Departamento de Entomologia e Acarologia, Piracicaba, São Paulo, Brasil; 2 Universidade de São Paulo, Faculdade de Filosofia, Ciências e Letras, Departamento de Química, Ribeirão Preto, São Paulo, Brasil; 3 Universidade Estadual de Campinas, Instituo de Biologia, Departamento de Biologia Animal, Campinas, São Paulo, Brasil; Universidade Federal do Rio de Janeiro, BRAZIL

## Abstract

The exploration of new niches for microorganisms capable of degrading recalcitrant molecules is still required. We hypothesized the gut microbiota associated with insect-resistant lines carry pesticide degrading bacteria, and predicted they carry bacteria selected to degrade pesticides they were resistant to. We isolated and accessed the pesticide-degrading capacity of gut bacteria from the gut of fifth instars of *Spodoptera frugiperda* strains resistant to lambda-cyhalothrin, deltamethrin, chlorpyrifos ethyl, spinosad and lufenuron, using insecticide-selective media. Sixteen isolates belonging to 10 phylotypes were obtained, from which four were also associated with the susceptible strain. However, growth of gut bacteria associated with larvae from the susceptible strain was not obtained in any of the insecticide-based selective media tested. Growth of isolates was affected by the concentration of insecticides in the media, and all grew well up to 40 μg/ml. The insecticide-degrading capacity of selected isolates was assessed by GC or LC-MS/MS analyses. In conclusion, resistant strains of *S*. *frugiperda* are an excellent reservoir of insecticide-degrading bacteria with bioremediation potential. Moreover, gut-associated bacteria are subjected to the selection pressure imposed by insecticides on their hosts and may influence the metabolization of pesticides in insects.

## Introduction

Several fitness traits of insects are heavily influenced by associated microbiota [1–3]. The association of insects with microbials is very important for the evolution of ecological features and/or feeding habits in insects. Symbiotic-associated polydnavirus aids in host exploitation by parasitoids [4], and flagellates, bacteria and yeasts allow insects to feed on hard to digest and/or nutritionally-poor diets [5]. Although insects may be associated with a variety of microbials, associations with bacteria are the most spread and common. More we investigate on the role of bacterial associations with insects, more we learn on how diverse their contribution to insects is. While contribution of primary, obligate insect-associated bacteria is basically related to nutrient provisioning to the host insect, secondary, facultatively insect-associated bacteria may influence a range of fitness traits of their host insect, including host nutrition. Secondary bacterial symbionts improve host immune response to entomophages [6–8] and entomopathogens [9–11], influence host plant selection [12], provide protection against heat stress [13], and contribute to the process of detoxification of metabolites produced for plant defense against herbivores [14–16]. The role of microbiota in detoxification of xenobiotics also includes the catabolization of organic molecules employed in applied pest control, as demonstrated by degradation [17,18] or histochemical analyses [14,19].

The diverse role and effect of symbionts to insect fitness traits has now gained new perspectives for biotechnological exploration. Insect associated symbionts are currently investigated *i)* for the development of new strategies for pest control and insect-vector disease management [20–23], and *ii)* as a source of molecules for biotechnological applications [24,25], such as enzymes for biomass conversion [26,27], antimicrobial and insecticide bioactive molecules [28,29], and molecules to manipulate insect behavior [30,31], for example.

Nonetheless, the participation of the microbiota associated with insects in the degradation of xenobiotics, including the synthetic organic compounds generally used for pest control raises important issues. The first would be related to the possibility to explore insect associated microbiota to select for bacteria capable to degrade xenobiotics for further application in bioremediation. Secondly, the role of microbiota in influencing the efficiency of insecticides in insect pest control [32–34].

Microorganisms have been widely used for environmental decontamination and are quite common in areas that have been contaminated by improper and/or excessive use of pesticides in agricultural areas [35–37]. Such intensive and continuous use of organic pesticides also produces another undesirable effect, as it leads to the evolution of insect resistance to insecticides [38,39]. Insecticide resistance always leads to a surge in pesticide use, resulting in increased side-effects to non-target organisms and environment [40,41]. As conservation and sustainable agricultural practices are progressively demanded by society, there is a constant need for bioactive molecules and/or microorganism that could be employed in the biodegradation of such xenobiotics. Bacteria have been exploited as one of the main targets for the development of biotechnological products that could be used in such applications, and contaminated soils are usually the source of potential bacterial candidates for bioremediation of contaminated areas [42–45].

The exploration of new niches as sources of new microorganisms or microorganisms with higher potential for degradation of xenobiotics remains a necessity. Insects share a long history of association with bacteria, and there are a number of indications insect-associated microbiota participate in the degradation of natural [14] and synthetic organic molecules [14,17,46,47]. Besides, the gut microbiota of insects has been shown to be amenable to community changes in response to pesticides [48] and evolution of pesticide resistance [49]. As current data have demonstrated that phenotypes are the result of the expression of the genomes of all organisms living in a close association [50,51], we hypothesized that laboratory insect strains selected for insecticide resistance would also carry bacteria selected to degrade the pesticides their hosts were selected against, and as such serve as potential new sources for the target-oriented selection of bacteria for bioremediation applications.

## Materials and methods

### Insects

Field populations of *Spodoptera frugiperda* have been exposed to F2 screening procedure for selection of genotypes and establishment of resistant lines [52] to pyrethroid (deltamethrin and lambda-cyhalothrin), organophosphate (chlorpyrifos ethyl), naturalyte (spinosad), and to chitin synthesis inhibitor (lufenuron). All of the resistant lines of *S*. *frugiperda* have been selected and constantly maintained under selection pressure in the Arthropod Resistance Management Laboratory, Department of Entomology and Acarology (ESALQ/USP). Lines were reared on a bean based artificial diet under controlled conditions (27 ± 1°C; 60 ± 10% RH; 14 h photophase), following standard rearing procedures [53].

### Isolation of gut bacteria

Fifth instars of *S*. *frugiperda* from each resistant line available (deltamethrin, lambda-cyhalothrin, chlorpyrifos ethyl, spinosad and lufenuron) and the susceptible reference line were removed from their rearing containers, surface-sterilized in 5% sodium hypochlorite in 70% ethanol for 5 min, and rinsed in sterile distilled water (3x—1min). Larvae were dissected in minimum medium 9 (MM9) [54] under sterile conditions, and the gut obtained was rinsed in clean, sterile MM9. Guts from each line were individually macerated in 1 mL MM9 in plastic tubes with the aid of sterile pestles, and 6 guts were later pooled, resulting in a 6 mL sample. Samples were vortexed and the volume of 1 gut/equivalent was transferred to 6 mL of MM9 added of 10 μg/mL of the insecticide the strain had been selected against in 0.1% Tween 20 solution (hereafter denominated as the selective medium) (Insecticide purity: deltamethrin = 98.5%; lambda-cyahalothrin = 87.4%; Chlorpyriphos = 99.2%; Lufenuron = 99.7%; Spinosad = 95.5%). Two selective media were prepared for each insecticide. One containing only the insecticide as carbon source for bacterial growth, while the other was supplemented with 1g/L glucose. Glucose was initially used to provide an additional nutritional carbon source for fastidious bacteria.

Samples were incubated at 28°C under constant shaking (225 rpm) for up to 10 days. Aliquots were removed at 5, 7 and 10 days after the beginning of the cultivation. Serial dilutions were prepared (1, 1/10, 1/100) and aliquots plated on MM9 agar supplemented with 10 μg/mL of the original insecticide. Samples were incubated at 28°C for bacterial growth and colony isolation. Experiments were run in triplicates. Bacterial colonies were isolated based on their morphology (size, color, and shape) and growth. At least three colonies per morphotype were selected and subjected to reisolation before molecular characterization. Each one of the selected colonies were grown in liquid medium (MM9 + 10 μg/mL insecticide) at 28°C under constant shaking (225 rpm) for 5 days. Samples were subjected to centrifugation, cell pellets were resuspended in MM9 and most of the cells were stored in 20% glycerol at -80°C. The remaining cells were centrifuged (2000g x 5 min) and used for genomic DNA extraction.

### Molecular identification of bacterial isolates

Bacterial cells were subjected to genomic DNA (gDNA) extraction following Sunnucks & Hales (1996) [55], and gDNA quality was verified by gel electrophoresis in a 0.8% (w/v) agarose gel slab containing 0.5 μg/mL ethidium bromide in TAE buffer (40 mM Tris-acetate, 1 mM EDTA, pH = 7.2) at constant voltage (70 V) for 1 h. gDNA samples were also evaluated by UV measurements and the OD ratio at 260/280 nm determined.

An almost complete sequence of the 16S rRNA gene was used for the molecular identification of the isolates obtained. 10–20 ng gDNA of each isolate was subject to PCR amplification using the universal primer set 8F (5’-AGAGTTTGATCCTGGCTCAG-3’) and 1491R (5’-GGTTACCTTGTTACGACTT-3') [56] in 1x enzyme buffer, 1.5 mM MgCl_2_, 200 μM dNTPs, 320 pM of each primer, and 0.625 U of Taq polymerase (Promega), in a final reaction volume of 25 μL. PCR cycling conditions were 4 min at 95°C (1x); 95°C—1 min, 55°C—1 min, and 72°C—2 min (35x), followed by a final extension at 72°C for 10 min (1x). Amplicons were resolved by gel electrophoresis in a 1% agarose slab as described before. Amplicons were visualized using an UV transilluminator coupled to an image acquisition and processing device (DNR Bioimaging Systems). PCR reactions were subjected to standard ExoSap treatment (Thermo Scientific, USA) (1U exonuclease and 1U alkaline phosphatase) for elimination of residual dNTPs and primers. Samples were incubated at 37°C for 30 min, and then at 80°C for 15 min.

Aliquots (4 μL) of the amplicons were first subjected to restriction fragment length polymorphism (RFLP) analysis using *EcoRI*, *Rsa* or *DdeI* (Promega) in a reaction volume of 10 μL (5 U of enzyme). Samples were incubated at 37°C for 4h, and the restriction pattern observed after gel electrophoresis in 1.2% agarose as before. Restriction fragment sizes were calculated after image acquisition and processing using the GelQuant software (DNR Bio-Imaging Systems Ltd.). Amplicons that yielded the same restriction pattern for the same morphotype were grouped and two amplicons selected for bidirectional sequencing. Amplicon sequencing was conducted at the Human Genome Studies Center (CEGH), University of São Paulo. Amplicons were subjected to sequencing using the primers set of the original amplification and the internal universal primer 515F (5'-GTGCCAGCAGCCGCGGTAA-3') to obtain the complete sequence [57].

Sequences obtained were analyzed using FinchTV v1.4.0 (Geospiza Inc.) and different reads were assembled in a single sequence using the Blast2 tool. After sequence trimming, 1350 bp were selected for heuristic blast search against databases at the “National Center for Biotechnology Information” (NCBI) (http://www.ncbi.nlm.nih.gov/) and EzTaxon-e (http://eztaxon-e.ezbiocloud.net/) [58] for putative identification of the isolates obtained against reference sequences.

Phylogenetic analysis of the sequences obtained for the different isolates was obtained after alignment using *ClustalW* as implemented in Mega 6.0, with a gap penalty = 15 and gap extension = 9 [59] (Tamura et al, 2013). Phylogenetic relationships were inferred by Neighbor-Joining [60] to obtain a phylogenetic tree, and the Jukes and Cantor model [61] used to calculate the distance matrix. 16S rDNA sequences of bacteria closely related to those we isolated from the gut of *S*. *frugiperda* were obtained from the NCBi/EzTaxon-e databses and used for alignment.

### Detection of isolated strains in susceptible larvae

As no bacterial colonies from the gut of the susceptible line of *S*. *frugiperda* grew on insecticide-based selective media, the occurrence of the selected isolates in the gut of the susceptible larvae was checked by diagnostic PCR.

Fifth instars of the susceptible line of *S*. *frugiperda* were dissected as before, the guts of five larvae were pooled and DNA extracted using the DNeasy Blood & Tissue kit (QIAGEN). gDNA qualitative and quantitative evaluation were done as before. Diagnostic PCR amplifications were carried out using 10–20 ng gDNA, 1x enzyme buffer, 1.5 mM MgCl_2_, 200 μM dNTPs, 320 pM of each primer (Table 1), and 0.625 U of Taq polimerase (Promega), in a final reaction volume of 25 μL. Thermocycling conditions were 1 cycle at 95°C for 4 min, followed by 35 cycles at 95°C for 1 min, X°C (given by each primer set) for 1 min, 72°C for 2 min, and a final extension at 72°C for 10 min. Amplicons were resolved in 1% agarose gel electrophoresis as before. Amplicons from positive amplifications were treated with exonuclease and alkaline phosphatase and subjected to bidirectional sequencing as earlier described.

**Table 1:** Species-specific primer sets, annealing temperature and expected amplicon size (pb) used in diagnostic-PCR amplifications for detection of gut microbes isolated from resistant larvae of *Spodoptera frugiperda* in the gut of a susceptible strain.

Symbiont	Primer (5’– 3’)	Annealing (°C)	Amplicon (bp)
*Enterococcus casseliflavus*^abcd^	Ec-1: ATGGAAGAAAGTTGAAAGGCG Ec-2: AAGGGGAAGCTCTATCTCTAG	56	820
*Enterococcus mundtii*^abde^	Em-1: ATGGTTTCGTTTTGAAAGGCG Em-2: AGGGGTGAACAGTTACTCTC	56	285
*Delftia lacustris*^a^	Da-1: ACTGGTTGTTGGGAATTAGTTTTC Da-2: TGTGTGCAGGTTCTCTTTC	56	211
*Leclercia adecarboxylata*^a^	La-1: ATCAGATGTGCCCAGATGG La-2: CAAGGGAACAACCTCCAAG	58	601
*Microbacterium paraoxydans*^a^	Mp-1: ATACTGGATATGTGACGTGAC Mp-2: ACTAGTTCCCAACGTTTACG	56	644
*Pseudomonas stutzeri*^b^	Ps-1: GCAGTAAGTTAATACCTTGCTG Ps-2: ACCCTCTGCCATACTCTAG	56	196
*Arthrobacter nicotinovorans*^c^	An-1: TCAACTCCGGTTCTGCAGT An-2: AGGTCTTTCCGGTTCATGTC	56	374
*Pseudomonas psychrotolerans*^*d*^	Pp-1: AGGAAGGGCTCATAGCGAATACC Pp-2: CTACCGCACTCTAGCCAGACA	58	197
*Microbacterium arborescens*^e^	Ma-1: GGTCAGTAGCTGGAAAGAAT Ma-2: GTTTCCAGACGTTTCCTCTATA	56	804
*Staphylococcus sciuri* subsp. *sciuri*^e^	Ss-1: TCGGCTGTCACTTATAGATGG Ss-2: CCGTCAAGACTTGTTCAGTTA	56	254

Superscript letters indicate the insecticide the host strain was resistant to: ^a^chlorpyrifos ethyl, ^b^lambda-cyhalothrin, ^c^deltamethrin, ^d^spinosad and ^e^lufenuron.

### Bacterial growth on insecticide-based media

The growth of bacterial isolates in insecticide-based growth media was investigated to select the most suitable bacteria for each insecticide for further analysis on insecticide degradation. Bacterial growth curves were assessed by inoculating 120 μL 10^5^ CFU/mL in 3 mL MM9 supplemented with 10 μg/mL of insecticide, under constant shacking as before. The colony forming units (CFU) was assessed by daily plating 10 μL of serial dilutions of 100 μL aliquots, followed by incubation at 28°C for 48 h. Isolates were cultivated for up to 5 days. Each treatment was replicated three times and each sampling time was done in technical triplicates.

### Bacterial growth under different concentrations of insecticides

Dose-dependent studies of the effects of insecticides on the growth of the isolated bacteria were conducted only for the best growing bacteria obtained from the previous experiment. Selected isolates were grown in MM9 media with different concentrations of insecticides (10, 20, 40, 80, and 160 μg/mL). Samples were prepared and bacterial growth assessed as earlier described for 5 days of cultivation or until no changes in the number of viable cells were observed.

### GC-MS and LC-MS/MS analyses

Insecticide degradation by the selected microbiota was investigated by cultivating 200 μL 10^5^ CFU/mL of each isolate in vials with 50 mL MM9, each containing 10 μg/mL of the insecticide tested. Samples were grown at 28°C under constant shaking as before. Each treatment was replicated three times. Samples of the growth media without bacteria were maintained under the same conditions to allow the assessment of natural degradation of the tested insecticides.

After cultivation, samples were centrifuged (10000g x 10 min x 4°C), the supernatant collected and subjected to liquid-liquid extraction using an equal volume of ethyl acetate (HPLC grade—JTBaker). Samples were vigorously mixed, the organic phase collected and concentrated in a rotary evaporator (40 rpm; 45°C). The residues obtained were recovered and stored in amber vials at -20°C for further GC or LC-MS/MS analyses depending on the chemical nature of each insecticide.

Samples obtained from cultures using the pyrethroids deltamethrin and lambda-cyhalothrin were dried under N_2_, recovered in 200 μL MeOH and injected in a HP6890 gas chromatograph equipped with a 30 m long x 320 μm id HP-5MS (5% diphenyl 95% dimethylpolisiloxane) capillary column (Agilent 19091J-413), coupled to a HP5973 mass detector operated in the electron impact mode at 70eV. Samples (1 μL) were injected in split-mode (20:1) using He as the carrier gas at a flow rate of 1.2 mL/min, and 250°C at the injection port. Oven temperature was initially set at 200°C followed by 4°C increases/min until 300°C, followed by a 5 min hold at 300°C. Identification of the target ions were made by comparisons to the fragmentation pattern obtained with the use of technical products as standards—deltamethrin [*m/z* 172 (30), 175 (29), 181 (100), 208 (15), 209 (16), 253 (57)] and lambda-cyhalothrin [*m/z* 181 (100), 197 (92), 208 (72), 449 (10)]. Deltamethrin and lambda-cyhalothrin concentrations in the samples were calculated using serial dilutions (50, 200, 350, 500, and 650 μg/mL) for determination of standard curves (deltamethrin: y = 145788x, *r*^*2*^ = 0.998; lambda-cyhalothrin: y = 245320x, *r*^*2*^ = 0.998).

Samples for chlorpyrifos ethyl, lufenuron and spinosad were obtained as before but were subjected to analysis using a liquid chromatograph ACQUITY UPLC *H-Class* coupled to a XEVO TQ-S mass spectrophotometer (Waters, Manchester, UK). The system was equipped with a quaternary pump, a vacuum degas system, and an automatic injector. Analyte chromatography separation was obtained using an ACQUITY UPLC BEH C_18_ column (50 mm long x 2.1 id; particle size = 1.7 μm) using acidified water (A: 0.1% ammoniun acetate in water) and methanol (B: 0.1% formic acid in MeOH) as mobile phases, starting with 35%A:65%B and ending with 3%A:97%B for a running time of 5 min at a flow rate of 0.4 mL/min. Mass spectrum acquisition data occurred with 3.2 kV capillary voltage, source temperature 150°C, N2 desolvation temperature 350°C, cone gas flow 150 L/h. Sample ionization was obtained using a Z-spray ionization source operated in positive (chlorpyrifos ethyl and spinosad) and negative (lufenuron) modes, and ion monitoring followed the multiple reaction monitoring strategy (MRM). Argon (Ar) was used as the collision gas for molecule dissociation (CAD—collision activated dissociation). Identification was done by comparisons with the fragmentation pattern produced by using technical products. Chlorpyrifos ethyl—parental ion *m/z* 350, daughter ion *m/z* 97, 350 > 97. Spinosad—spinosyn A: parental ion *m/z* 773, daughter ion *m/z* 142, 773 > 142; spinosyn D—parental ion *m/z* 747, daughter ion *m/z* 142, 747 > 142. Lufenuron—parental ion *m/z* 509, daughter ion *m/z* 326, 509 > 326. The concentration of these compounds was calculated using standard curves based on serial dilutions curves (0, 5, 10, 50, 100 e 250 ng/mL)—chlorpyrifos ethyl: y = 2E+07x, *r*^*2*^ = 0.999; Spinosad: spinosyn A: y = 5E+07x, *r*^*2*^ = 0.997, and spinosyn D: y = 1E+07x, *r*^*2*^ = 0.996; and Luneuron: y = 2E+06x, *r*^*2*^ = 0.978). All data acquisition for LC-MS/MS analysis was done using the MassLynx 4.1 software and the application manager for TargetLynx (Waters).

MS and MS/MS data obtained were used to determine the changes in the concentration (%BD) of each insecticide tested remaining available in suspension after bacteria cultivation. The %BD was given by subtracting the total degradation in the sample (%TD) by the natural degradation observed in the control (%ND). All samples were run in biological triplicates and averages within each treatment were compared by a *t* test (*p* < 0.05).

## Results

### Selective isolation of insecticide-degrading bacteria

Supplementation of the basal medium MM9 with insecticides was very efficient to sustain the growth of gut bacteria adapted to such substrates as their nutritional resource from the gut of resistant lines of *S*. *frugiperda*, but no bacterial isolates were obtained from the gut of susceptible larvae (S1 Fig). Moreover, our data also indicate the selection pressure imposed on insects by their continuous exposure to xenobiotics can also works as a selective pressure acting upon on the gut microbiota of *S*. *frugiperda*. No bacterial growth was obtained from cultivation of the gut microbiota of susceptible larvae of this species, even though the microbiota of resistant and susceptible larvae share different phenotypes of identical phylotypes. Phylotypes that were common to the gut microbiota of insecticide-resistant and susceptible strains of *S*. *frugiperda* only grew on insecticide-based media if coming from the gut of the resistant line. Moreover, common phylotypes from resistant strains only grew on the selective media based on the insecticide their host strain was selected against. In other words, bacterial isolates reflected the phenotypes of the strains of *S*. *frugiperda* they were associated with. We also demonstrated that lines resistant to different insecticides carry particular gut symbionts capable of exploring the source insecticide as a food resource.

More than 120 bacterial morphotypes were isolated from the gut of *S*. *frugiperda* larvae resistant to insecticides (S1 Fig). Successful isolation of bacteria from the gut microbiota associated to insects resistant to deltamethrin only occurred in MM9 supplemented with glucose (S1 Fig). Glucose supplementation also improved selective isolation of bacteria from the gut of lines resistant to chlorpyrifos ethyl and spinosad. On the other hand, microbiota from the gut of lines resistant to lambda-cyhalothrin or lufenuron grew better in glucose-free media. Bacteria from lines resistant to pyrethroids grew faster in glucose-free media (S2 Fig). In spinosad and lufenuron, isolates were obtained after prolonged cultivation in liquid media (S2 Fig). Time of cultivation in liquid media did not affect the number of isolates obtained from the gut microbiota of larvae resistant to deltamethrin (S2 Fig).

### Molecular characterization of gut-associated insecticide-degrading microbiota

Molecular analysis of all morphotypes by PCR-RFLP followed by sequencing of the 16S rDNA led to the identification of five phylotypes from the microbiota of chlorpyrifos ethyl resistant larvae (IIL-Cl 05, 22, 25, 29 and 32), two from deltamethrin (IIL-Dm 01 and 05), and three each from lambda-cyhalothrin (IIL-Lc 09, 16 and 32), spinosad (IIL-Sp 06, 16 and 32) and lufenuron (IIL-Luf 12, 14 and 18). Heuristic comparative searches using 1350 bp of the 16S rDNA of these isolates led to their putative identification (Table 2).

**Table 2:** Putative identification of bacterial isolates obtained from the larval gut of strains of *Spodoptera frugiperda* resistant to different insecticides.

Isolated	Similarity		
	Nearest match	GenBank accession	Identity (%)
IIL-Cl05 (KX280768)^a^	*Enterococcus casseliflavus*	AJ420804	99.6
IIL-Cl22 (KX273063)^a^	*Delftia lacustris*	EU888308	99.8
IIL-Cl25 (KX280769)^a^	*Enterococcus mundtii*	AJ420806	99.9
IIL-Cl29 (KX272966)^a^	*Leclercia adecarboxylata*	AB273740	99.9
IIL-Cl32 (KX280770)^a^	*Microbacterium paraoxydans*	AJ491806	99.8
IIL-Lc09 (KX280771)^b^	*Pseudomonas stutzeri*	CP002881	99.9
IIL-Lc16 (KX280772)^b^	*Enterococcus mundtii*	AJ420806	99.9
IIL-Lc32 (KX280773)^b^	*Enterococcus casseliflavus*	AJ420804	99.6
IIL-Dm01 (KX280774)^c^	*Enterococcus casseliflavus*	AJ420804	99.6
IIL-Dm05 (KX280775)^c^	*Arthrobacter nicotinovorans*	X80743	99.4
IIL-Sp06 (KX280776)^d^	*Enterococcus casseliflavus*	AJ420804	99.6
IIL-Sp19 (KX280777)^d^	*Pseudomonas psychrotolerans*	AJ575816	100.0
IIL-Sp24 (KX280778)^d^	*Enterococcus mundtii*	AJ420806	99.9
IIL-Luf12 (KX280779)^e^	*Staphylococcus sciuri* subsp. *sciuri*	AJ421446	99.9
IIL-Luf14 (KX280780)^e^	*Microbacterium arborescens*	X77443	99.9
IIL-Luf18 (KX280781)^e^	*Enterococcus mundtii*	AJ420806	99.9

Identification is based on 16S rDNA (1350 pb) sequence similarity obtained from heuristic search against sequences in NCBi and EzTaxon-e databases. Superscript letters indicate the insecticide the host strain was resistant to: ^a^chlorpyrifos ethyl, ^b^lambda-cyhalothrin, ^c^deltamethrin, ^d^spinosad, and ^e^lufenuron.

**Fig 1:**
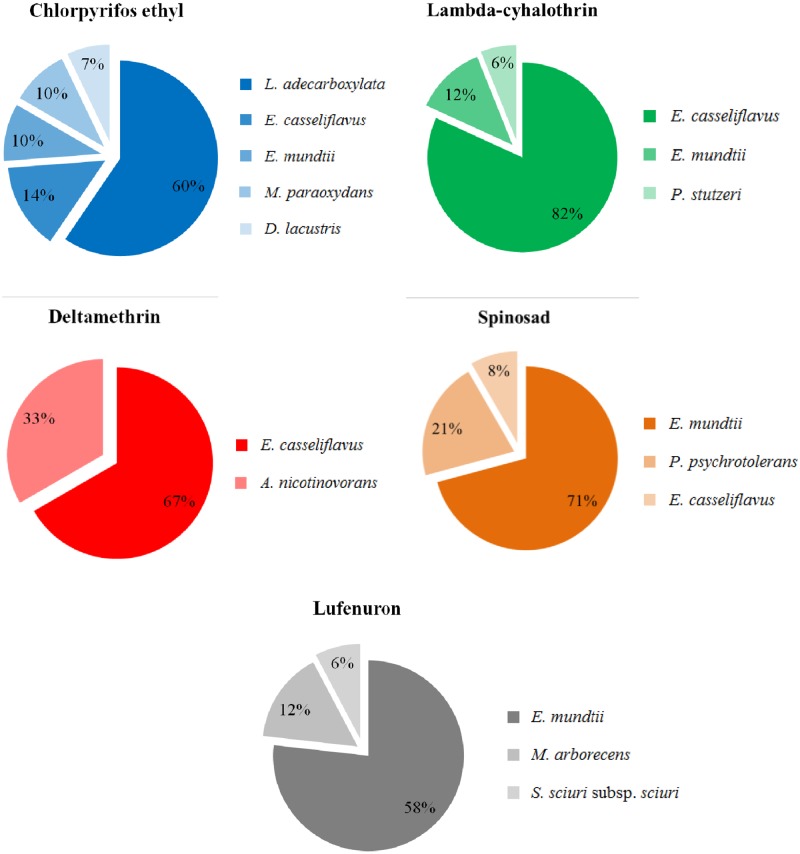
Relative proportion (%) of phylotypes isolated from the larval gut of insecticide-resistant lines of *Spodoptera frugiperda* afterr RFLP-PCR analysis and partial sequence (550 pb) of the 16S rDNA. Putative identification obtained after heuristic search of 1350 bp of 16S rDNA against sequences available in the NCBi and EzTaxon-e databases.

**Fig 2:**
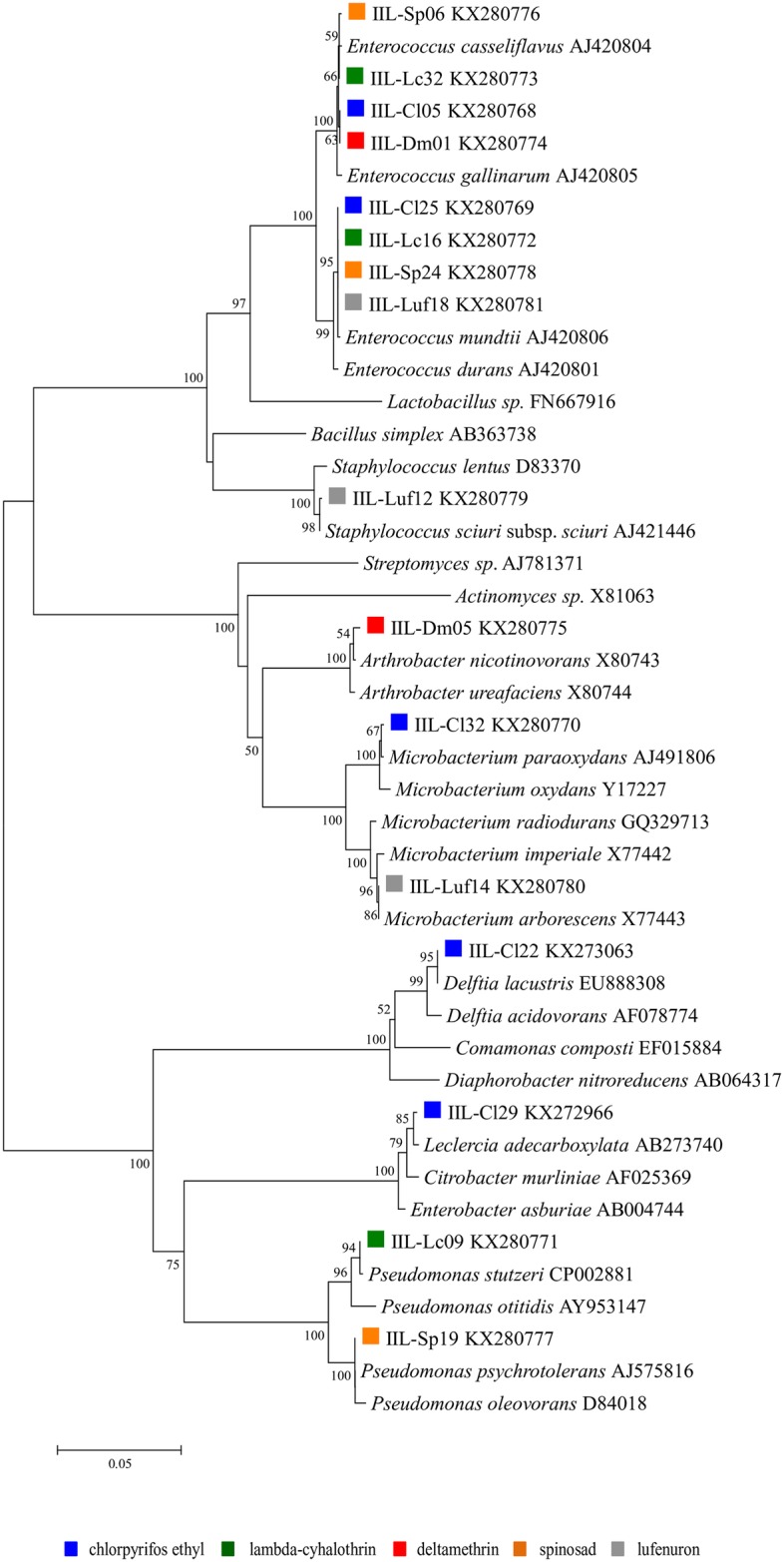
Neighbor-joining phylogenetic tree of bacterial isolates obtained from the larval gut microbiota of insecticide-resistant lines of *Spodoptera frugiperda*. Tree was built using 1350 bp sequences of the 16S rDNA. Support bootstrap values are shown in the branches. Scale bar indicates 0.02 substitutions per nucleotide position.

The *Enterococcaceae Enterococcus casseliflavus* and *Enterococcus mundtii* were common to the microbiota of chlorpyrifos ethyl, lambda-cyhalothrin and spinosad. Yet, *E*. *mundtii* was isolated from lufenuron resistant larvae while *E*. *casseliflavus* from deltamethrin (Table 2, Fig 1). They were also the most abundant bacteria isolated for most of the selective media they grew, with the exception of chlropyrifos ethyl, in which *Leclercia adecarboxylata* dominated (Table 2, Fig 1). All remaining bacteria were exclusively selected on the selective media containing the insecticide the strain they were associated with was resistant against. *Microbacterium paraoxydans*, *Delftia lacustris* and *L*. *adecarboxylata* were only isolated from the gut of chlorpyrifos ethyl resistant larvae, *Pseudomonas stutzeri* from lambda-cyhalothrin, *A*. *nicotinovorans* from deltamethrin, *Pseudomonas psychrotolerans* from spinosad, and *Staphylococcus sciuri* subspecies *sciuri* and *Microbactetium arborescens* from lufenuron (Table 2, Figs 1 and 2).

Analysis of the phylogenetic tree generated with the sequences of the isolated strains and of type species indicated that almost all of the strains we isolated resolved in well-defined clades with their closest type species (Fig 2). Isolates originating from different host resistant strains putatively identified as *E*. *casseliflavus* and *E*. *mundtii* grouped in their specific clades, all with very high bootstrap support values (Fig 2).

### Detection of insecticide-degrading bacterial isolates

Although no isolates were obtained from the gut of susceptible larvae of *S*. *frugiperda* in selective media, diagnostic-PCRs detected *E*. *casseliflavus*, *E*. *mundtii*, *S*. *sciuri* and *P*. *stutzeri* in the gut of susceptible larvae. Identification of the isolates was obtained by sequencing the amplicons produced, which all shared 99.9% identity with their reference sequence.

### Growth of insecticide-degrading bacteria

**Fig 3:**
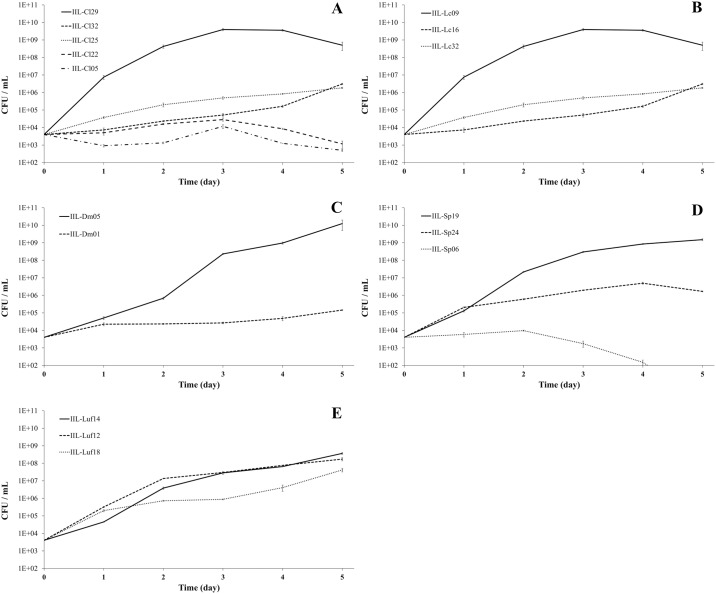
Growth of insecticide-degrading bacterial isolates obtained from the gut microbiota of strain of *Spodoptera frugiperda* resistant to the insecticides chlorpyrifos ethyl (A), lambda-cyhalothrin (B), deltamethrin (C), spinosad (D), and lufenuron (E) when cultured in minimum medium M9 added of 10 μg/mL of the insecticide the host insect strain was resistant to.

The growth capacity of the insecticide-degrading bacteria in the selective media containing the insecticide they were selected against indicated isolates IIL-Cl29 (*L*. *adecarboxylata*) (10^9^ CFU/mL in 3 days), IIL-Lc09 (*P*. *stutzeri*) (10^9^ CFU/mL in 5 days), IIL-Dm05 (*A*. *nicotinovorans*) (10^10^ CFU/mL in 5 days), IIL-Sp19 (*P*. *psychrotolerans*) (10^9^ CFU/mL in 5 days), IIL-Luf14 (*M*. *arborescens*) (10^8^ CFU/mL in 5 days) grew better and faster in selective-media based on chlorpyrifos ethyl, lambda-cyhalothrin, deltamethrin, spinosad and lufenuron, respectively (Fig 3). Isolates obtained from the gut of larvae resistant to several insecticides, such as *E*. *mundtii* e *E*. *casseliflavus*, were not as competitive to grow on insecticide-based media (Fig 3).

**Fig 4:**
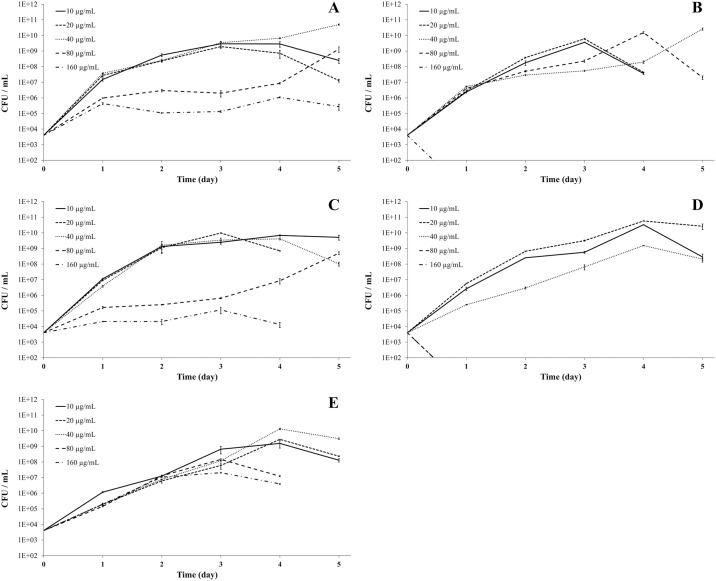
Growth of selected insecticide-degrading bacterial isolates obtained from the gut microbiota of insecticide-resistant lines of *Spodoptera frugiperda* at different insecticide concentrations (10, 20, 40, 80 or 160 μg/mL). Isolates A) IIL-Cl29 (*Leclercia adecarboxylata*), B) IIL-Lc09 (*Pseudomonas stutzeri*), C) IIL-Dm05 (*Arthrobacter nicotinovorans*), D) IIL-Sp19 (*Pseudomonas psychrotolerans*), and E) IIL-Luf14 (*Microbacterium arborescens*) were isolated and cultivated on insecticide-based media containing, respectively, chlorpyrifos ethyl, lambda-cyhalothrin, deltamethrin, spinosad and lufenuron.

Isolates with the best growth performance were further selected to investigate their growth response under different concentrations of insecticide. Most of them had a similar growth trend up to 40 μg/mL of insecticide in their growing media. With the exception of *P*. *psychrotolerans*, all other bacteria had some growth in media with 80 μg/mL. But none grew in concentrations as high as 160 μg/mL (Fig 4).

### Insecticide use by selected insecticide-degrading bacteria

GC-MS or LC-MS/MS analysis of growth media indicated the selected isolates obtained from the gut of insecticide-resistant lines of *S*. *frugiperda* cleared from 27 to 77% of the insecticide they were selected against during the period of cultivation tested (Table 3). Natural loss of the insecticides by exposure to the environment ranged from 0 to 9%, with lambda-cyhalothrin being the most sensitive to natural degration (Table 3). Chlorpyrifos ethyl was the least used (27%), while spinosad, mainly the spinosyn D fraction, was the most used by bacteria (77%) (Table 3).

**Table 3:** Natural (ND), bacterial (BD) and total (TD) degradation of lambda-cyhalothrin, deltamethrin, chlorpyrifos ethyl, lufenuron and spinosad (spinosyn A e D) after 5 days of cultivation of bacterial isolates from the larval gut of insecticide-resistant lines of *Spodoptera frugiperda*.

Insecticide	Isolate	Degradation (±SE, n = 3)		
		TD %	ND %	BD %*
Lambda-cyhalothrin	IIL-Lc09 –*P*. *stutzeri*	46.5 ± 7.2	9.3 ± 3.7^b^	37.2 ± 3.9^a^
Deltamethrin	IIL-Dm05 –*A*. *nicotinovorans*	59.6 ± 6.1	4.7 ± 4.6^b^	54.9 ± 9.3^a^
Chlorpyrifos ethyl	IIL-Cl29 –*L*. *adecarboxylata*	31.0 ± 9.8	4.0 ± 1.2^b^	27.0 ± 8.7^a^
Lufenuron	IIL-Luf14 –*M*. *arborescens*	38.0 ± 16.7	0.0 ± 0.0^b^	38.0 ± 16.7^a^
Spinosyn A	IIL-Sp19 –*P*. *psychrotolerans*	48.6 ± 10.3	2.8 ± 1.4^b^	45.8 ± 11.6^a^
Spinosyn D		81.0 ± 7.2	3.8 ± 1.9^b^	77.2 ± 9.2^a^

*BD% = TD%—ND%; means followed by different letters within lines indicates the bacterial degradation differs from the natural degradation using *t* test (*p*≤0.01)

## Discussion

We demonstrate the gut of insecticide-resistant lines of *S*. *frugiperda* is a successful environment for the isolation of insecticide-degrading bacteria, revealing an underexplored niche for the search of microbials targeted to biodegradation applications. All selected bacteria demonstrated continuous growth up to 40 μg/mL, a concentration that is much higher than the CL95 of some of the insecticides tested (e.g. spinosad and lufenuron—CL95 = ~2 μg/mL) and close to the CL50 of others (e.g.lambda-cyhalothrin—CL50 = ~55 μg/mL), demonstrating they would be exposed and able to metabolize the concentrations applied in the field to control *S*. *frugiperda*.

Degradation of organophosphates has been reported for *Delftia acidovorans* [62], *Microbacterium* [63] and *Pseudomonas* [64], but little is available on the microbial degradation of the organophosphate chlorpyrifos ethyl. *Arthrobacter* sp. [65], *Enterobacter asburiae* [66], and the *Pseudomonadaceae Pseudomonas stutzeri* [45] and *Pseudomonas kilonensis* [67] are the few microbials reported to degrade chlorpyrifos-ethyl. We identified a new potential candidate for bioremediation of contaminated areas with chlorpyrifos-ethyl, as there are no records for *Leclercia adecarboxylata* degrading this insecticide. As far as we know, *Arthrobacter nicotinovorans* and *Pseudomonas stutzeri* are also new records of microbials with potential to degrade deltamethrin and lambda-cyhalothrin, respectively, even though their potential in bioremediation has been acknowledged [68,69].

Spinosad and lufenuron degradation in the soil is based on microbial activity [70,71], but no potential microbials that degrade these compounds have yet been identified. Our data clearly demonstrates the gut microbiota associated with insects resistant to insecticides offers a rich environment for the isolation of potential microbials for bioremediation against target compounds.

The association of different bacterial species with the gut of insecticide resistant lines of *S*. *frugiperda* able to grow on selective media based on insecticides the host insect was resistant against indicates the gut microbiota of *S*. *frugiperda* is also under pressure during the directed selection of resistant lines. Such differences also suggest that insect populations exposed to different selection pressures under natural conditions may contribute with a diversified microbiota. Moreover, the occurrence of the same microbial species in most of the resistant and susceptible lines of *S*. *frugiperda*, but with different potential to explore the insecticides tested as nutritional resources demonstrate gut microbials are also subjected to selection by the continuous exposure to insecticides. Bacteria that are exposed to directed selective pressures are of great interest because their genetic machinery has been selected to respond to the source of stress [72]. In the case of resistant insects, bacteria have to be able to metabolize the insecticides to survive, once several pesticides may also have antimicrobial activity [73,74]. But the gut microbiota is subjected to a number of stress factors that may alter its composition. Nutritional factors have been under intense investigation and there are clear evidence on the role the food source, genetics, and others factors play in shaping the gut microbiota [75,76], including that of insects [48,49,77].

The role of insect symbionts in the metabolization of xenobiotics is seldom reported [17,18,73], but several have already argued on their potential on the host nutritional ecology [5], enhancing the host immune system against microbials and macrobials [11,78], adaptation to the environment [79], insect-plant interactions [15] and on host population dynamics [80]. Thus, there have been few examples in which insect associated symbionts were demonstrated to increase the host tolerance to natural and synthetic stressors, including insecticides. The increased tolerance of insect to insecticides provided by microbial symbionts led to the suggestion that microbial symbionts may also contribute to the evolution of insect resistance to insecticides [47,81,82]. Gut-associated microbes are a valuable resource as they contribute to the overall nutritional ecology of the host by participating of processes involved in host food utilization (digestion, detoxification), nutrient recycling and nutrient provisioning, and interfere with the host multitrophic interactions [83].

Insects can use several strategies for detoxification of xenobiotics in the gut lumen by providing a reducing environment and a complex of enzymes (monooxygenases, esterases, hydrolases, transferases) to cleave or modify the target xenobiotic for excretion [84,85]. The relevance of the gut microbiota contribution to the enzymatic reactions that occur in the lumen of the host gut can be demonstrated by the use of microbiota-related enzymes to overcome enzyme inhibitors produced by plants the host insect feeds on [81,86,87]. There are indications the microbial enzymatic contribution in the gut lumen could also contribute to the degradation of insecticides ingested by the host, and the hydrolysis of these compounds would render nutrients for the microbiota growth [88]. The diversity and differences between prokaryote and eukaryote-produced enzymes indicate microbial enzymes could greatly contribute with the metabolization of insecticides in contaminated insects [34,89].

We demonstrated the gut microbiota of insecticide-resistant lines is a rich resource for the isolation of microbes capable to degrade insecticides, and a promising tool for biotechnological exploration in bioremediation programs [32–35]. However, the contribution of the associated microbiota to processes of detoxification of insects and in the level of tolerance or in the evolution of resistance to insecticides remains to be further investigated. Investigation should be concentrated in mechanisms of symbiont acquisition and in processes microbes employ to metabolize such substrates, as the successful contribution of gut microbes has been reported for a soil-acquired bacterium [47].

## Supporting information

S1 Fig Total bacterial morphotypes (128) isolated from the midgut of larvae of strains of *Spodoptera frugiperda* resistant to different insecticides, using the M9 minimum medium added of 10 μg/mL of the insecticides the strain was resistant to, supplemented (1 g/L) or not with glucose. (TIF)

S2 Fig Bacterial morphotypes isolated from the midgut of larvae of strains of *Spodoptera frugiperda* resistant to different insecticides, using the M9 minimum medium added of 10 μg/mL of the insecticides the strain was resistant to, supplemented (1 g/L) or not with glucose, after different periods of cultivation in liquid media. (TIF)

S3 Fig Standard curves for lambda-cyhalothrin and deltamethrin used in GC-MS analysis (50, 200, 350, 500 and 650 μg/mL), and for chlorpyrifos ethyl, lufenuron, spinosyn A and spinosyn D used in UPLC-MS/MS analysis (50, 100, 150, 200 and 300 ng/mL). (TIF)
