# Supplementary figures and images for: The gut microbiota of insecticide-resistant insects houses insecticide-degrading bacteria: A potential source for biotechnological exploitation
Source: PLoS One. 2017 Mar 30;12(3):e0174754. doi: 10.1371/journal.pone.0174754 (PMC5373613; doi:10.1371/journal.pone.0174754)

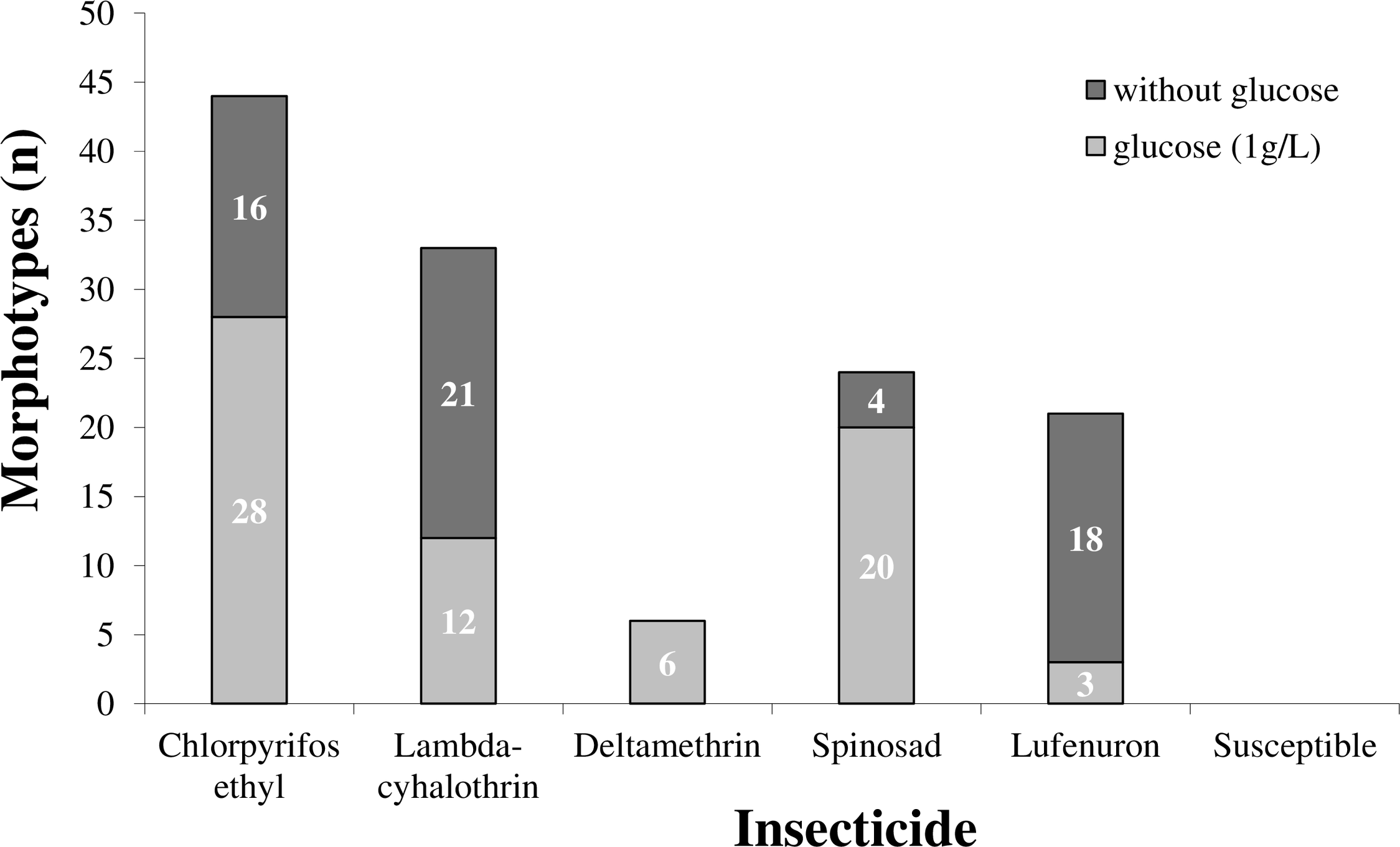

Supplement: S1 Fig — (TIF) [file pone.0174754.s001.tif]

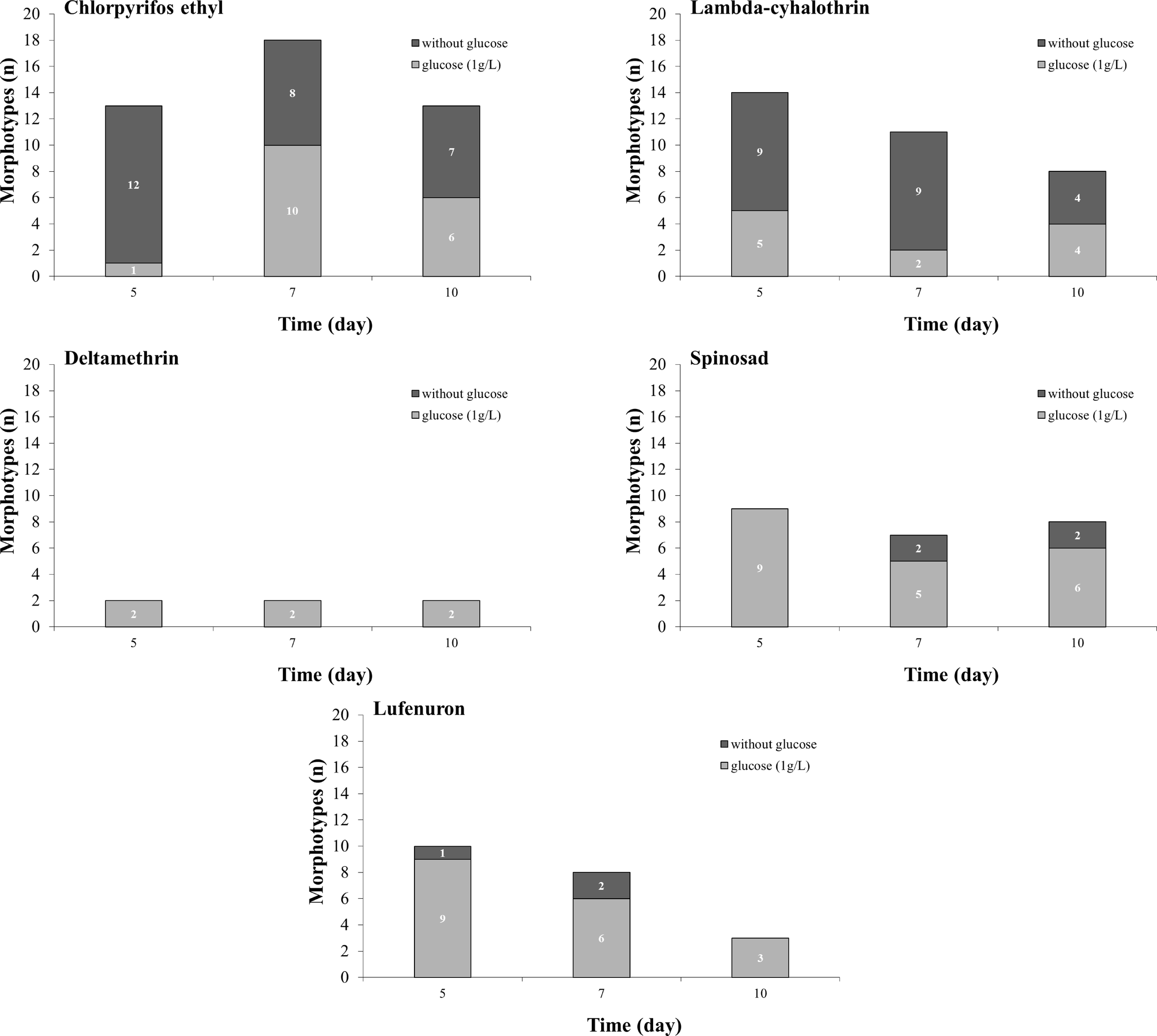

Supplement: S2 Fig — (TIF) [file pone.0174754.s002.tif]

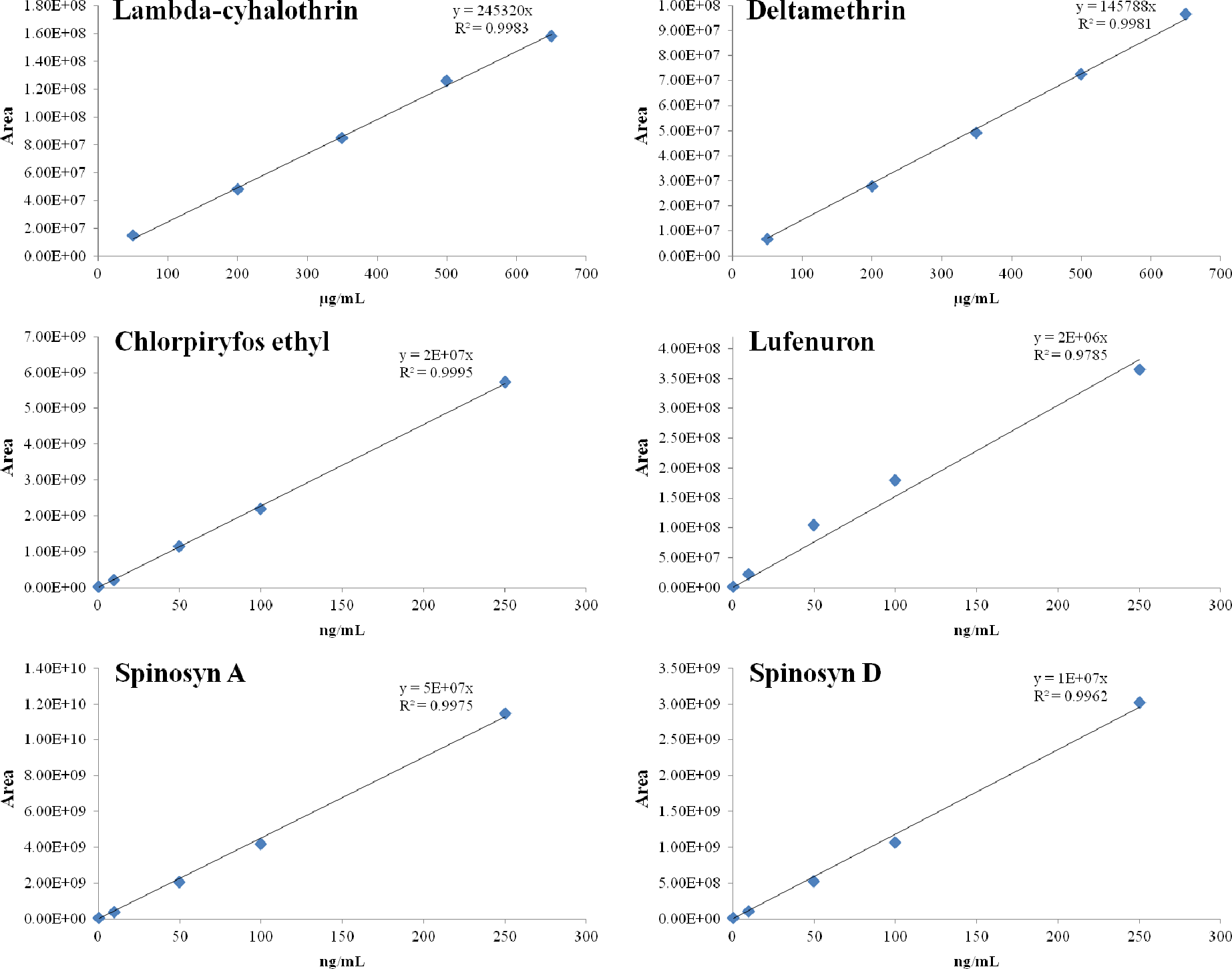

Supplement: S3 Fig — (TIF) [file pone.0174754.s003.tif]
